# Experimental Infection of Mink Enforces the Role of *Arcanobacterium phocae* as Causative Agent of Fur Animal Epidemic Necrotic Pyoderma (FENP)

**DOI:** 10.1371/journal.pone.0168129

**Published:** 2016-12-14

**Authors:** Heli Nordgren, Kirsi Aaltonen, Mirja Raunio-Saarnisto, Antti Sukura, Olli Vapalahti, Tarja Sironen

**Affiliations:** 1 FFBA Finnish Fur Breeders Association, Vantaa, Finland; 2 Fin Furlab (FFBA), Vaasa, Finland; 3 University of Helsinki, Department of Veterinary Biosciences, Faculty of Veterinary Medicine, Helsinki, Finland; 4 University of Helsinki, Department of Virology, Faculty of Medicine, Helsinki, Finland; 5 Evira, Finnish Food Safety Authority, Seinäjoki, Finland; 6 Department of Virology and Immunology, HUSLAB, Hospital district of Helsinki and Uusimaa, Helsinki, Finland; Instituto Butantan, BRAZIL

## Abstract

Fur Animal Epidemic Necrotic Pyoderma (FENP) is a severe, often lethal infectious disease affecting all three fur animal species: mink (*Neovision vision*), foxes (*Vulpes lagopus*) and finnraccoons (*Nyctereutes procyonoides*). Previous studies showed an association between *Arcanobacterium phocae* and FENP. An experimental infection was conducted to confirm the ability of *A*. *phocae* to infect mink either alone or concurrently with a novel *Streptococcus* sp. found together with *A*. *phocae* in many cases of FENP. Different inoculation methods were tested to study possible routes of transmission. Typical signs, and gross- and histopathological findings for FENP were detected when naïve mink were infected with the tissue extract of mink with FENP, using a subcutaneous/ intradermal infection route. Edema, hemorrhage, necrosis and pus formation were detected in the infection site. A pure culture preparation of *A*. *phocae* alone or concurrently with the novel *Streptococcus* sp. caused severe acute signs of lethargy, apathy and anorexia and even mortality. The histopathological findings were similar to those found in naturally occurring cases of FENP. In contrast, the perorally infected mink presented no clinical signs nor any gross- or histopathological lesions. This study showed that *A*. *phocae* is able to cause FENP. The study also indicated that predisposing factors such as the environment, the general condition of the animals, temperature and skin trauma contribute to the development of the disease.

## Introduction

Fur Animal Epidemic Necrotic Pyoderma (FENP) is a newly discovered, emerging disease which affects animals in the fur industry. At present, the etiology remains unconfirmed, but a strong correlation was found between *Arcanobacterium phocae (A*. *phocae*) and FENP. [1] The disease is known to affect the major species in this industry, including mink (*Neovison vison*), foxes (*Vulpes lagopus*) and Finnraccoons (a raccoon dog bred for fur, *Nyctereutes procyonoides*). In all species, FENP infection causes necrotic pyoderma, however the manifestations differ slightly between species. In mink, lesions are detected in the paws and head; in foxes, a conjunctivitis spreads to the eyelids and facial skin; and in raccoon dogs, abscesses develop in the paws [1]. The disease develops rapidly, has no effective treatment and has rapidly evolved to a severe animal welfare problem as well as cause of financial losses to the farmers and the fur industry.

Nordgren et al, (2014) detected the bacterium *A*. *phocae* in tissues of animals with FENP. In some cases it was found together with a novel *Streptococcus* sp. [1]. *A*. *phocae* is a gram positive, small, pleomorphic, coccobacillary rod. On blood agar it grows, within 24 hours, as small, pinpoint like colonies with a strong hemolytic zone around each colony. Cultivation in a CO^2^-enriched environment does not enhance growth. It is catalase-positive, oxidase-negative, and elicits a positive CAMP-reaction to *Rhodococcus equi* and *Streptococcus agalactiae*, and an inverse CAMP-reaction to *Staphylococcus aureus*. [2]

In addition to fur animals, *A*. *phocae* has been isolated from marine mammals, including harbor seals, common seals, dolphins, and sea lions. It is generally found in superficial mixed infections, abscesses and wounds. Many of the infections are secondary to skin tissue damage, such as bites, bullet wounds or other traumatic injuries in the skin tissue. [2, 3] Discovering *A*. *phocae* in tissues of animal showing signs of FENP, later in the text referred to as FENP affected animals, is particularly interesting as the onset of FENP related signs were first seen in mink fed with seal byproducts in the USA in 1970 and in Canada in 1996 [4]. The bacterium can also be found on Canadian farms suffering from a chronic pododermatitis but not on farms with healthy animals [5].

An experimental infection was performed to investigate the ability of *A*. *phocae* to infect mink and cause FENP either alone or concurrently with the novel *Streptococcus* sp. It is crucial to confirm the causative agent of this disease in order to develop appropriate diagnostics, prevention protocols, and future treatments.

## Materials and Methods

### Ethics statement

This study was performed in strict accordance with the Finnish Act on animal Experimentation 62/2006, with the European convention for the protection of vertebrate animals used for experimental and other scientific purpose (Directive 86/609/EEC) fully implemented. The housing and management of mink fulfilled all the requirements given to the housing of mink (VNa1084/2011). All the experimental procedures of the study were approved by the Animal Experiment board in Oulu (Permit Number: ESAVI/6780/04.10.03/2012). The animals were monitored regularly and euthanized when sever systemic signs such as fever, anorexia or apathy or inflammatory signs in the skin were detected to avoid unnecessary prolonged pain or distress and suffering. The animals were euthanized by CO gas, the method described in legislation concerning culling of the animals ((EU) N: o 1099/2009).

### Animals

Mink (*Neovison vison*) were bought from an ordinary fur farm since mink are not bred for experimental use. The farmer was informed of the experimental use of the purchased mink. The farm had no history of FENP, and the animals were free from plasmacytosis (Aleutian mink disease), which is a common viral mink disease that affects immunity and is suspected to predispose animals to other diseases. [6, 7] Black color type was selected because it was known to be susceptible to FENP. One hundred females, born the previous spring, were used in the experiment. The breeder females were selected for the experiment as there were no males left for sale during the winter season.

### The bacterial inocula

The inocula were prepared from cultures of *A*. *phocae* and the novel *Streptococcus* sp. isolated from clinical cases of FENP [1]. Six separate *A*. *phocae* isolates, two from each fur animal species, were used in mixed preparation to account for possible differences in virulence. Correspondingly a mixture of three separate isolates of the novel *Streptococcus* were used for the dual infections. The bacterial species were confirmed by sequencing a partial 16S rDNA gene (all nine isolates), and a partial 16S-23S ribosomal RNA intergenic spacer sequence of the three *Streptococcus* isolates. The unique sequences have been submitted to NCBI GenBank under the accession numbers KX966275-KX966278. The bacteria were grown over night on blood agar plates, containing 4% defibrinated sheep blood. They were scraped off the plates and suspended in injection grade saline solution. The concentration was determined by spectroscopy based on experiments and adjusted to set concentrations based on the viability experiment (described below). The bacteria remained in suspension for approximately five hours prior to infection.

To ensure the viability and infectivity of the bacterial strains to be used in the experiment, they were pretested by a six hour incubation in saline suspension at +4°C at different, known concentrations based on experiments correlating OD600 with cfu. The +4°C was used in the testing because of the subzero temperatures at the experimental farm. The suspensions, both pre- and post-incubation, were plated onto blood agar plates with 4% defibrinated sheep blood and incubated overnight at +37°C. The colonies were counted the following day.

In addition to pure cultures, tissue suspension from foot lesions of three FENP affected mink was used for inoculation of the experimental mink. These three animals were obtained from a farm with confirmed history of FENP. The diseased tissues were cut and homogenized with mortar and pestle, diluted in saline and filtered through a 5 μm syringe filter (Millex, Merc, Darmstadt, Germany). Samples of the diseased tissues used in the infection were taken for PCR studies (more detailed description by Nordgren et al [1]) and also plated onto blood agar plates to confirm the presence of the bacteria of interest. The doses in all the experiments remained in suspension for three to five hours which could cause a reduction in the amount of infectious bacteria which was accounted for in the amounts suspended.

### Experimental infections

The experimental infection was performed on a fur farm not used for housing mink at the time of the experiment. The farm had no previous history of FENP. The shelter building used in the experiment had been empty for 14 months and the cages were mechanically cleaned before the trial. The mink were transported to the experimental farm, caged, marked individually, and allowed to adjust to the environment for two weeks prior to the infections. One mink died during the adaptation period.

The experiment was performed from January to March, because clinical experience had shown that cold weather predisposes the animals to FENP. During the two pilot studies the temperature varied between 0°C and +4°C. The temperature dropped to -20°C during the main experiment. The winter season is optimal for an infection trial also because the density of the animals is at its lowest thus limiting the possibility of unintentional transmission.

Two pilot experiments were performed to determine the optimal dose and route for the main study; we used either peroral (p.o.) infection route or subcutaneous (s.c.)/intradermal (i.d.) injections to the right hind foot (Table 1). For the first pilot study thirty mink were used, and they were penned in one end of the shed. The trial lasted for four weeks. Four different doses were tested in the first trial, two for each infection route. The p.o. inoculum was mixed in feed either as a dose of 200 cfu), or a dose of (10 000 cfu) of *A*. *phocae*. The potential oral and/or intranasal infection routes were mimicked by infecting via feed. Both routes might be possible if feed were a source of infection. The mink were fed once a day and the infection suspension was mixed in the total feed portion. On the day of infection, a slightly smaller feed portion, approximately 150 g, was used to ensure that the mink ate the whole portion including the infection dose. The s.c./i.d. infection was performed by injecting 0.5 ml of either a dose of 50 cfu or 2500 cfu by a 20 G needle to the right hind leg. The mink were also given homogenized skin tissue filtrate from lesions of FENP affected mink via feed and as a s.c./i.d. injection. In addition, we performed p.o. and s.c./i.d. infections using tissue suspension from FENP affected animals. Control animals were fed (2) and injected s.c./i.d. (2) with the saline solution used for bacterial suspensions (9 mg/ml NaCl solution, Fresenius Kabi, Bad Homburg, Germany). Two mink were left as controls without any procedures to make sure that the group did not become infected from the environment. Animals were monitored signs of disease for at least twice a day during the experimental period.

**Table 1:** The design and results of the pilot experiments.

**Pilot 1**						
			**Findings in the necropsies (not done for all animals)**			
Bacteria in inocula	(Dose and) route	Clinical signs (n)/n	Typical gross pathology / no. necropsies	Typical histo-pathology / no. necropsies	*A*. *phocae* in culture / PCR / no. necropsies	Novel *Strept*. in culture / PCR /no. necropsies
Controls^A^	p.o. and s.c./i.d.	0/6	0/3	0/3	0/3/3	0/3/3
*A*. *phocae*	Low p.o. 200 cfu	0/4	ND	ND	ND	ND
*A*. *phocae*	High p.o. 10 000 cfu	0/4	0/1	0/1	0/1/1	0/1/1
*A*. *phocae*	Low s.c./i.d. 50 cfu	0/4	ND	ND	ND	ND
*A*. *phocae*	High s.c./i.d. 2500 cfu	0/4	ND	ND	ND	ND
FENP tissue	p.o.	0/4	0/1	0/1	0/1/1	0/1/1
FENP tissue	s.c./i.d.	1/4	1/2	1/2	0/2/2	1/2/2
		1/30	1/7			
**Pilot 2**						
Controls		0/2	ND	ND	ND	ND
*A*. *phocae*	Higher p.o. 9 x 10^6^ cfu	0/2	ND	ND	ND	ND
*A*. *phocae*	Higher s.c./i.d. 4 x 10^6^ cfu	2/2	2/2	2/2	2/2/2	0/2/2
mixture	p.o. 2 x 10^6^ cfu (each)	0/2	ND	ND	ND	ND
mixture	s.c./i.d. 2 x 10^6^ cfu (each)	2/2	2/2	2/2	2/2/2	2/2/2
		4/10	4/4			

^A^ Controls include two animals that were left completely untreated

ND, Not done

Based on the results of the first pilot study, we continued to a second pilot study to test higher doses (Table 1), 9 million cfu p.o. and 4 million cfu s.c./i.d. of *A*. *phocae*. We also used a mixture of 2 million cfu of *A*. *phocae* and 2 million cfu of the novel *Streptococcus*. In the second pilot study ten mink were used. A combination of *A*. *phocae* and the novel *Streptococcus* sp. was used in pilot study 2 because the results of the previous study of FENP (Nordgren et al 2014) had shown that especially in FENP affected mink *A*. *phocae* was often isolated in mixed cultures with a novel *Streptococcus* sp. Two mink were left as controls between pilot study animals and the rest of the study animals waiting for the main experiment. The second pilot study lasted for four weeks. The main study was designed based on the information obtained from these two pilot studies.

Based on the findings in the pilot infections, we proceeded to the main study in which we used 59 mink (Table 2). Blood serum samples were taken prior the infection by drawing blood from a clipped nail into a capillary tube containing heparin (VWR Microhematocrit Capillary Tubes, VWR, USA, PA). The blood samples were tested by PCR for *A*. *phocae* and the novel *Streptococcus* sp. after the experiment. Blood samples instead of swabs were taken because the blood samples could be used later on to detect other causative agents, such as viruses, the possible role of which is yet unknown in pathogenesis of FENP.

**Table 2:** Design and results of the main experiment.

				Findings in the necropsies (not done for all animals)			
group	Bacteria in inocula	(Dose and) route	Clinical signs (n)/n	Typical gross pathology / no. necropsies	Typical histo-pathology / no. necropsies	*A*. *phocae* in culture / PCR^A^ / no. necropsies	Novel *Strept*. in culture / PCR^A^ /no. necropsies
	Controls		0/11	ND	ND	ND	ND
**A**	*A*.*phocae*	p.o. 5 x 10^6^ cfu.	0/6	0/5	0/5	0/3/5	0/2/5
**A**	mixture	p.o. 5 x 10^6^ cfu (each)	0/6	ND	ND	ND	ND
**B**	*A*. *phocae*	Low s.c. 3 x 10^5^ cfu	6/6	6/6	6/6	5/5/6	0/2/6
**B**	*A*. *phocae*	High s.c. 3 x 10^6^ cfu	5/6	5/5	4/5	4/5/5	0/5/5
**C**	*A*. *phocae*	Low, trauma 3 x 10^5^ cfu	5/6	5/5	5/5	5/5/5	0/4/5
**C**	*A*. *phocae*	High, trauma 3 x 10^6^ cfu	5/6	5/6	5/6	4/6/6	0/6/6
**D**	mixture	trauma 5 x 10^5^ cfu (each)	5/6	5/6	5/6	3/4/6	4/4/6
**E**	FENP tissue	s.c.	3/6	3/4	3/4	0/4/4	3/2/4
			29/59	29/37			

^A^ PCR was only done on samples with inconsistencies with expected results

The p.o. infection route by feed was substituted by direct application onto the facial area and mouth. In the pilot studies it was difficult to mix the infection dose in feed in the cold circumstances, and thus the actual dose eaten was uncertain. The animals were divided into five groups A-E. Group A was infected by injecting one ml of the *A*. *phocae* and the novel *Streptococcus* sp. suspension (5 000 000 cfu each) or pure *A*. *phocae* at 5 000 000 cfu suspension with a syringe to the nasal area and mouth of the mink. Group B received a dose of 300 000 cfu or a dose of 3 000 000 cfu of *A*. *phocae* s.c./i.d. in 0.5 ml of saline solution. This was to test the ability of *A*. *phocae* alone to cause the typical signs of FENP. Group C received either the 300 000 cfu or the 3 000 000 cfu dose to a skin wound artificially created by scraping the area with a scalpel. This route caused more tissue damage than s.c./i.d. injections alone, thus better mimicking a trauma to the skin, which was earlier noted to predispose the animals to FENP. Group D mink received a mixture of *A*. *phocae* (500 000 cfu) and novel *Streptococcus* sp. (500 000 cfu) in an artificial skin wound (as in Group C) to further test the possible synergy of the two bacteria. In Group E mink were injected s.c./i.d. with tissue suspension from three FENP affected mink. An 18 G needle was used to facilitate the passing of the suspension, thus creating a larger than normal injection trauma.

Artificial skin wounds were also created in six control mink and one mink was left completely untouched. In addition, four mink were transferred to the cages of animals that died/were euthanized during the second pilot test to study a possible infection route by contact with the cage of a diseased animal.

Aliquots of the inocula were plated on blood agar plates after the infection to confirm the cfu. The plates were then taken to a laboratory and incubated overnight at +37°C. PCR and subsequent sequencing was done on the tissues from diseased animals used in the experiment to confirm the presence of *A*. *phocae* and the novel *Streptococcus* sp.

### Signs of disease and gross pathology

All animals showing systemic signs or signs of inflammation in the skin were immediately euthanized and necropsied. At the end of the experiment all remaining animals were euthanized and selectively necropsied. The clinical signs of FENP, in the acute form, were determined to be general signs of illness i.e. fever, malaise, anorexia and lethargy combined with one or more of the specific signs of FENP. These were considered to be discharge from the eyes and nose, swelling of the foot and avoidance to use the limb followed by skin lesions in the feet and/or facial area. The lesions were considered specific for FENP when they were exudative, necrotic and a typical crust could be detected. The gross lesions were described by the location, extension and severity (mild, moderate, severe) of the lesion and the duration (acute, chronic) of the process. Any lesions in the internal organs were also recorded.

### Histopathology

Samples of brain, heart, lung, trachea, spleen, liver, kidney, bladder, duodenum, jejunum, ileum, colon, local lymph nodes and skin tissue were fixed in 10% phosphate buffered formalin for at least 24 h, embedded in paraffin and sectioned at 4 micrometers. All tissues were stained with hematoxylin and eosin (H&E). All the animals with clinical signs were submitted to a complete necropsy; also selected controls (4) and infected animals without signs were fully necropsied (9). Samples of brain, heart, lung, spleen, liver, kidney, duodenum, jejunum, ileum, colon, local lymph nodes and skin were sent for bacteriological studies and cultured on blood agar plates containing 5% defibrinated sheep blood and incubated aerobically at 37°C for 24–48 hours. Intestinal samples were cultured to detect salmonella, campylobacter and anaerobic pathogens. Bacteriological culturing was performed on all fully necropsied animals.

### PCR

PCR tests (as described by Nordgren et al. 2014) were performed on the blood samples gathered prior to the infection. The samples were probed for the presence of both *A*. *phocae* and the novel *Streptococcus* sp. Samples taken at necropsy of infected and non-infected animals were tested by PCR if the results of either the infection or the bacterial cultures showed inconsistencies with the inoculated bacteria, and also selected samples from animals showing signs. The feed used in the experiment was also tested for the presence of the experimentally inoculated bacteria.

## Results

### Experimental infection protocol

In this experiment both pure cultures of the bacteria and tissue suspensions were used to infect animals. The culture suspensions of *A*. *phocae* and the novel *Streptococcus* sp. suspected to be associated with FENP in mink were first quantified by plating and counting colony forming units (cfu) to ensure the quality of the inocula after dilution and processing and storage times at ambient temperatures. The inocula were successfully adjusted to account for the lowered viability. The tissue suspension from animals with FENP which was used as an inoculum for the experimental infections, was confirmed to contain both *A*. *phocae* and the novel *Streptococcus* sp. by PCR and sequencing. The feed used in the experiment was found to contain DNA of Streptococcal (*Streptococcus agalactiae*) and Arcanobacterial (*Trueperella bernardiae*, previously known as *Arcanobacterium bernardiae*) origin but not the species used in the experiment. When plated the feed did not produce colonies except those expected to be found in the environment.

The PCR tests done on the pre-infection blood samples taken from the animals before the main experiment, showed that one mink had been exposed to *A*. *phocae* and another one to the novel *Streptococcus* before infection. The first mink, predisposed to A. phocae, belonged to group A and received *A*. *phocae* and the *Streptococcus* p.o. The *Streptococcus* carrier belonged in to group E which received the tissue suspension from a diseased animal.

### Clinical outcome

In the first pilot study, one mink infected s.c./i.d. with FENP- tissue suspension, developed a typical FENP lesion in the injection site. The lesion was a 0.5x0.5 cm necrotic pyoderma with brownish exudate and crust formation. All the other animals (29/30) remained healthy (Table 1). In the second pilot, all mink infected s.c./i.d. with a higher dose of *A*. *phocae* or a mixture of *A*. *phocae* and the novel *Streptococcus* sp., had severe systemic signs of lethargy, anorexia and apathy, and pyoderma with edema, hemorrhages and necrosis in the injection site. Two of the animals died of sudden, peracute infection before euthanasia could be performed. In the main study, almost all (26/30) of the animals inoculated either s.c./i.d. or via the artificial wound (groups B, C, D) developed a lesion in the inoculation site, as did half of the mink (3/6) inoculated with tissue of FENP affected animals (Table 2.). Mink inoculated p.o. did not develop FENP in any of the substudies, neither did any of the control animals.

### Pathology

**Fig 1:**
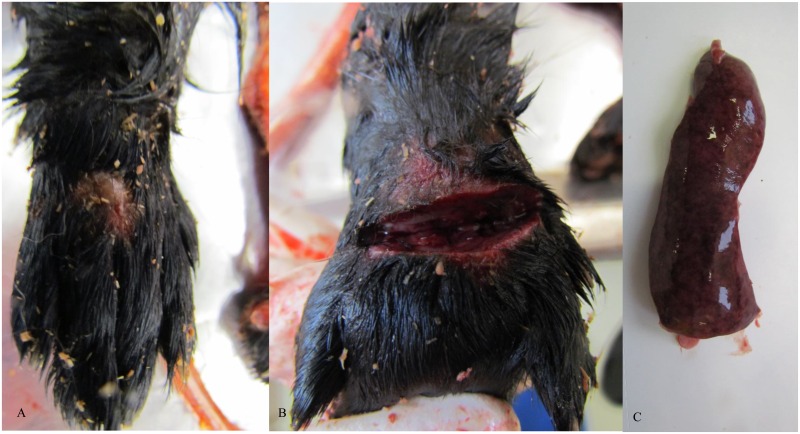
Gross pathological changes in infected mink. (A) Crust formation was detected in i.d./s.c. inoculation site with *A*. *phocae*. (B) A incision made in the inoculation site of i.d./s.c. inoculation with *A*. *phocae* and novel *Streptococcus* sp. revealed oedema, necrosis and haemorrhages and pus formation in the skin and in the subcutaneous tissues. (C) Enlarged spleen with mottled surface was detected in mink inoculated with both *A*. *phocae* and novel *Streptococcus* sp. Splenomegaly is a typical change in clinical cases of FENP.

**Fig 2:**
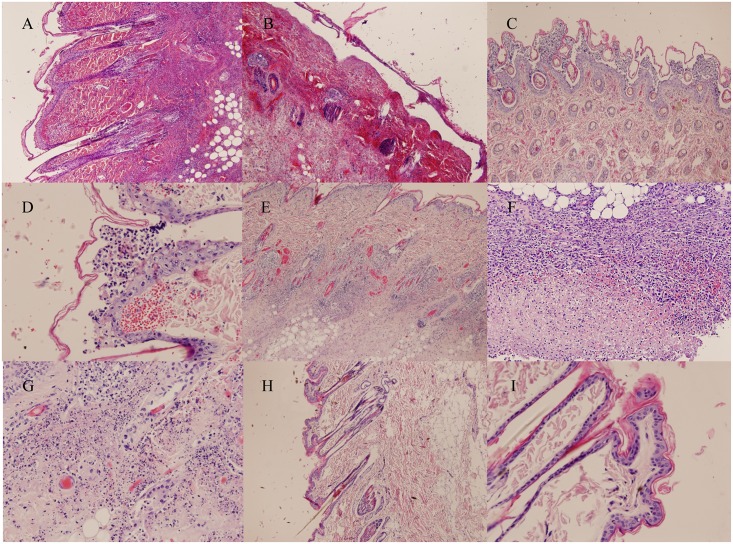
Histopathological findings in infected mink. (A) A section from the inoculation site in the left hind leg in mink inoculated with *A*. *phocae* and novel *Streptococcus* sp. s.c./i.d. shows acute, severe superficial and deep, diffuse neutrophilic inflammation. (B) Severe haemorrhage and diffuse neutrophilic inflammation is seen in the inoculation site in mink inoculated solely with *A*. *phocae* s.c./i.d. (C/D) A skin section from the mink inoculated with *A*. *phocae* and novel *Streptococcus* sp. shows sub corneal pustular inflammation with haemorrhages. (E) More deep diffuse neutrophilic inflammation is demonstrated in picture E from skin section from the mink inoculated with *A*. *phocae* s.c./i.d. (F) Severe necropurulent inflammation is seen in mink inoculated s.c./i.d. with tissue extract of FENP affected mink. (G) Similar inflammation is seen in mink inoculated with *A*. *phocae*. (H/I) Skin section from the control mink injected s.c./i.d. with NaCl solution showed no histopathological lesions.

Mink with clinical signs (34) in the pilot studies and the main study presented edema in the inoculation site, and brownish exudate on the skin. Mink from the first pilot inoculated with FENP tissue and mink from the second pilot with an artificially created wound had crust formation typical to FENP as well (Fig 1). Edema, hemorrhage, necrosis and pus were detected in the subcutaneous tissues (Fig 2). The spleens were dark red, enlarged (approx. four times the size of a normal spleen) and the surfaces were slightly mottled in 26/34 cases. Slight fatty liver changes were observed in 26/34 mink.

Mink with clinical signs and gross pathological findings (33/34) had histopathological changes in the skin samples as well. Lesions were similar as those seen in clinical cases of FENP. In the epidermal layers an acute inflammation was observed; necrosis, fibrin, clusters of gram positive bacteria and mainly diffuse neutrophilic inflammation were recorded. Inflammation spread to subcutaneous tissue, where hemorrhage, necrosis and edema were detected. Vasculitis and subcorneal micropustular inflammation were also occasionally detected. Hemorrhages, necrosis and fibrin in the spleen samples were detected in 28/34 mink, 19/34 had mild to moderate vacuolar degeneration in liver samples typical of fatty liver and 16/34 had congestion and moderate perivascular lympho- and plasmacytic inflammations and 11/34 fibrin and neutrophilic granulocytes in the lung sample. Mink inoculated s.c./i.d. with tissue of FENP affected animals (1 from pilot test, 3 from main study) developed histopathological lesions in the skin similar to those seen in FENP. Three mink inoculated with the tissue of FENP affected mink (1 p.o. and 2 s.c./i.d.) without clinical signs were necropsied and did not show any macroscopic changes. Three controls and the mink which died in the pre-experiment adaption period were necropsied as healthy controls. Six mink inoculated with *A*. *phocae* perorally and one inoculated through wounded skin showed no signs of disease and were selected for necropsy. One mink inoculated with both *A*. *phocae* and the novel *Streptococcus* sp. also failed to show clinical signs and was necropsied as well. No histopathological changes were detected in any of these animals.

### Microbiology

In the second pilot study and the main study, 23 mink inoculated with *A*. *phocae* developed skin lesions, and from 17 of these, *A*. *phocae* was isolated from the skin sample in mixed culture and from two as a pure culture. Four had bacterial growth with *A*. *phocae* in the internal organs (liver, kidney) as well. The isolation of *A*. *phocae* was unsuccessful in four mink that were inoculated with *A*. *phocae* s.c./i.d.. Two of these had *Proteus sp*. overgrowth and two had abundant growth of bacteria belonging to the *Staphylococcus intermedius* group. One inoculated animal that remained asymptomatic had *A*. *phocae* growth in the skin sample.

Seven out of eight mink inoculated with a mixture of *A*. *phocae* and the novel *Streptococcus* developed systemic signs and skin lesions. Both of the bacteria were grown from a skin sample of one of these mink, *A*. *phocae* alone from two, *Streptococcus* alone from three mink. Those mink which were inoculated but did not develop visible systemic signs or skin lesions had growth of both bacteria in the injection site as well. Four animals inoculated with both bacteria had the novel *Streptococcus* in the internal organs (kidney, spleen and liver) alone, and in one case with *Escherichia coli*.

Altogether three (3/6) mink inoculated with FENP affected tissue developed systemic signs and skin lesions, and the novel *Streptococcus* sp. together with *Streptococcus canis* was isolated from the skin samples of all these animals, and one also had *Proteus* sp.. Two of these had bacterial growth in internal organs (liver, kidney, and lung) as well *(the novel Streptococcus*, *E*.*coli*, *S*. *canis*, *Proteus* sp.). In the lung neutrophilic granulocytes and fibrin were detected and the liver showed mild steatosis. *A*. *phocae* could only be detected by PCR (see below)

Samples from the skin and organs of three healthy controls were cultured with no microbiological findings. The mink that died in the pre-experiment adaption period and six animals inoculated without signs were sent for necropsy, but no bacteria were isolated.

### PCR

The PCR tests performed on the pre-inoculation blood samples of the main experiment showed that one mink had been exposed to *A*. *phocae* and another one to the novel *Streptococcus* before the inoculation. The first mink, pre- exposed to *A*. *phocae*, belongs to group A and received *A*. *phocae* and the *Streptococcus* p.o. The *Streptococcus* carrier belonged to group E which received the tissue suspension from a diseased animal. While other animals belonging to these groups developed clinical signs, these two remained healthy.

The animals inoculated but not showing signs of infection and negative control animals were found to be negative or the copy numbers in PCR were extremely low (4 negative controls). Two mink had completely eliminated even the genetic material derived from the inoculum. The three mink inoculated with both bacteria and presenting with clinical signs but found positive only for the novel *Streptococcus* sp. by culturing were found positive for *A*. *phocae* by PCR.

## Discussion

Here we describe the setup of an experimental infection model for FENP by inoculation of mink with *A*. *phocae* and a novel *Streptococcus* sp. In our previous report on the disease, [1] an association between FENP and *A*. *phocae* and to a smaller extent *Streptococcus* spp. was detected. Our experimental infection model showed that tissue suspension from a FENP affected mink and *A*. *phocae* either alone or together with the novel *Streptococcus* sp. can cause lesions and signs which mimic those detected in FENP. These were observed in the animals inoculated by both s.c./ i.d. route and inoculation of artificially wounded skin. These findings resemble the infections detected in the marine mammals where *A*. *phocae* causes inflammation in wounds [2]. No signs or lesions were detected in the groups inoculated through the p.o. route however some of these animals were PCR positive, which suggests that feed may be one of the routes by which the bacteria colonize the animals, but this alone is not enough to induce disease. The mink transferred to the cages that had housed animals showing signs of FENP also remained healthy, again suggesting that environmental exposure alone is not sufficient to cause clinical disease. These animals were not tested by PCR so it remains unclear if they were colonized without any detectable signs of disease. All of this indicates that trauma, stress or an underlying infection predisposes to FENP and reinforces the previous theory of a multifactorial disease.

An ordinary mink farm was chosen for the experiment because no experimental laboratory premises suitable for mink existed in Finland. By this approach, we also replicated natural conditions on the farms, which was considered an advantage since practical experience suggests that environmental factors may predispose to the infection. On the other hand, a farm is suboptimal for an infection experiment because it compromises the biosafety of the experimental environment. The bacteria may be able to spread in the greater farm area much as in natural cases of FENP despite measures taken to prevent this. Indeed, the PCR results of the negative controls suggested that the bacteria had been able to spread within the experimental group of animals, supporting the high potential for transmission and rapid spread of these pathogens.

In the main study three out of six mink inoculated with tissues of an animal with FENP developed macroscopic lesions and histopathologic changes typical for FENP. One mink developed chronic lesions more typical for the FENP detected in clinical cases. The macroscopic signs and histologic lesions of FENP are distinct from other bacterial skin infections. Other factors separating FENP are the localization, early clinical signs, necrosis and severity of the lesions and also the epidemic nature of this disease. The bacteriological studies revealed the novel *Streptococcus* in all cases but no presence of cultivable *A*. *phocae*. All the cultures were mixed, and as *A*. *phocae* is easily overgrown by other bacteria it may be missed in conventional diagnostics. The PCR showed the presence of both bacteria in the animals with typical lesions. Animals inoculated with direct injection of *A*. *phocae*, a mixture of the two bacteria or the tissue, but showing no signs of the disease were negative for *A*. *phocae* both by culture and PCR showing that the PCR positive results were unlikely to come from the inoculum and were more likely indicative of bacterial growth in the tissues.

*A*. *phocae* has been detected by PCR in samples of clinically healthy animals [5] indicating that it is an opportunistic pathogen and can be part of the normal flora of the animals, causing problems only in favorable circumstances, for instance in the case of trauma to the skin or in the presence of other pathogens. The colonization of the fur animals appears to be recent as no isolates of *A*. *phocae* were found prior to emergence of FENP further suggesting a link between this bacteria and the disease. It is of course possible, if unlikely, that the bacteria could have gone undiagnosed until the arrival of FENP. In this study two animals shown to be carriers prior to the main experiment, had actually been housed close to the pilot animals suggesting a possible source of infection at the site of the experiment. These animals also remained healthy despite experimental infection which might suggest immunological protection stemming from previous low level exposure. Samples from control animals at the end of the experiment were PCR positive at low quantities for both bacteria whereas samples from control animals in the pilot studies were not. This suggests that the bacteria started to spontaneously spread among the test animal population correlating with the suspected, highly contagious nature of the bacteria. This could also explain the positive findings of the *Streptococcus* in experimental animals inoculated solely with *A*. *phocae*.

The inoculation doses were determined and adjusted in the pilot studies 1 and 2. Suboptimal doses were probably used in pilot test 1 as no signs or lesions were detected, whereas the relatively high dosage in the pilot test 2 caused severe systemic signs of apathy, lethargy and anorexia as well sudden mortality. The clinical picture was subacute to acute and no chronic lesions developed apart from one mink inoculated with tissue suspension of mink with FENP. The doses used for inoculation should be further optimized, as the clinical signs varied between subacute and severely acute causing deaths or leading to euthanasia. Sudden mortality is reported in natural cases of FENP and was seen also in the study animals. In such cases *A*. *phocae* is mostly isolated in the skin samples and no growth of microbes is detected in the organ samples. Even though the clinical outcome differed in severity and duration from the typical, more chronic, cases of FENP, both the gross and histopathological findings in the study animals including pyoderma and profound necrosis in the skin resembled the findings typical for FENP.

The difficulty to reach correct dosing was partly attributable to the poor viability or longevity of the bacteria in suspension. The viability is reduced in direct correlation with time of incubation in saline solution and also correlates with temperature which is more difficult to standardize in field conditions. Therefore, the amount of infectious bacteria may have varied within the experiment. The growth of *A*. *phocae* is relatively poor in laboratory conditions in general further complicating diagnostic approaches during experiments and to a lesser degree in natural cases of FENP as the sample material is prone to environmental contaminants.

The mink used in the study were in excellent health and the environment and management of the animals was good. This probably influenced the general immunological status of the animals in the beginning of the trial. During the main study weather conditions changed notably, with a sudden cold weather period with -20°C temperatures, which may have stressed and weakened the mink thus resulting in the signs observed during the main experiment.

It may be hypothesized that the systemic signs and lesions detected in diseased and succumbed animals could be explained with the effect of bacterial toxins. Bacteria belonging to genus *Arcanobacterium* are known to produce toxins with dermonecrotic activity, which could explain some of the features of the pathogenesis of FENP [8]. The presence and role of such toxin(s) requires further investigations, also the possible role of the Streptococci remains unclear. Bacteriological examinations of naturally occurring cases of FENP can show *A*. *phocae* alone or together with a *Streptococcus*, including the novel *Streptococcus* species that is closely related to Streptococci of marine origins. Our experiment enforces somewhat the hypothesis that the two bacteria have synergy but that the disease does not necessarily require the presence of the *Streptococcus* to develop. Further studies are under way to characterize and define the role of the *Streptococcus*. Additionally, more research should be done on the differences of viability and infectivity of different bacterial isolates and to overcome their poor longevity in saline solutions at low ambient temperatures. This is unlikely to be a characteristic of the wild type bacteria as they were originally found in marine mammals.

This study demonstrated that tissue suspension of a mink with FENP, *A*. *phocae* alone and together with the novel *Streptococcus* sp. cause lesions and mortality in mink. There were similarities between the signs and lesions as well as the gross and histopathological findings in the study animals with those typical to FENP, even in the more acute forms of the disease developed by the study animals. The study confirmed that predisposing factors like skin trauma and environmental factors are needed to evoke the disease.
